# 4-1BB-4-1BBL *cis*-interaction contributes to the survival of self-reactive CD8^+^ T cell

**DOI:** 10.1038/s41423-023-01056-3

**Published:** 2023-06-26

**Authors:** Eunjung Cho, Rohit Singh, Chungyong Han, Seon-Hee Kim, Kwang H. Kim, Bo-Mi Park, Dong Hoon Shin, Seongeun Han, Young H. Kim, Byoung S. Kwon, Ki Taek Nam, Beom K. Choi

**Affiliations:** 1 Immuno-oncology Branch, Division of Rare and Refractory Cancer, National Cancer Center, Goyang, 10408 Republic of Korea; 2 Severance Biomedical Science Institute, Brain Korea 21 Project for Medical Science, Yonsei University College of Medicine, Seoul, Republic of Korea; 3 Department of Cancer Biomedical Science, Graduate School of Cancer Science and Policy, National Cancer Center, Goyang, 10408 Republic of Korea; 4 Department of Biomedical Laboratory Science, Catholic Kwandong University, Gangneung, 25601 Republic of Korea; 5 Biomedicine Production Branch, Research Institute, National Cancer Center, Goyang, 10408 Republic of Korea; 6 Anticancer Resistance Branch, Division of Rare and Refractory Cancer, Research Institute, National Cancer Center, Goyang, 10408 Republic of Korea; 7 Eutilex Co., Ltd., Geumcheon-gu, Seoul, 08594 Republic of Korea; 8 Department of Medicine, Tulane University Health Sciences Center, New Orleans, LA 70112 USA; 9 Innobationbio Co., Ltd., Mapo-gu, Seoul, 03929 Republic of Korea

**Keywords:** Immunotherapy, Tumour immunology, Tumour immunology

4-1BB is an inducible receptor expressed on activated T cells, while its ligand, 4-1BBL, is mainly expressed in antigen-presenting cells and macrophages [1, 2]. To the best of our knowledge, ligand-mediated transactivation of 4-1BB is responsible for the survival and immune effector functions of T cells. However, there have been reports of 4-1BBL also being expressed in T cells [3, 4]. As 4-1BB has only one known ligand, 4-1BBL, this coexpression of 4-1BB and 4-1BBL on T cells raises questions about *cis*-interactions among the proteins and their potential role in T-cell activation and immune function. Therefore, to investigate whether 4-1BB and 4-1BBL signals are necessary to suppress tumor growth, we designed an experiment as shown in Fig. 1a. In vivo administration of the 3H3 clone strongly triggers 4-1BB signals along with no or minor 4-1BBL signals, TKS-1 induces a mild agonistic effect on 4-1BBL with blockade of 4-1BB signals, 17B5 blocks both 4-1BB and 4-1BBL signals, and coadministration of 3H3 and TKS-1 enhances both 4-1BB and 4-1BBL signals [5–7]. C57BL/6 mice received mAbs intraperitoneally every 5 days starting from 9 days after MC38 tumor injection (Fig. 1a). Compared to rat IgG treatment, 17B5 treatment did not affect the tumor growth rate, but it did increase mortality (Fig. 1b). While TKS-1 treatment alone was ineffective, the combination treatment of 3H3 and TKS-1 effectively suppressed tumor growth (Fig. 1b). The percentage and absolute number of tumor-specific pGP70^+^CD8^+^ T cells were significantly higher in the inguinal tumor-draining lymph nodes (iTDLNs) of mice treated with 3H3 alone or with TSK-1 in combination with 3H3 than in those treated with rat IgG, TKS-1, or 17B5 alone (Fig. 1c, d). The results suggest that the combination of 3H3 and TKS-1 enhances tumor-specific CD8^+^ T-cell responses in MC38 tumor-bearing mice.

**Fig. 1:**
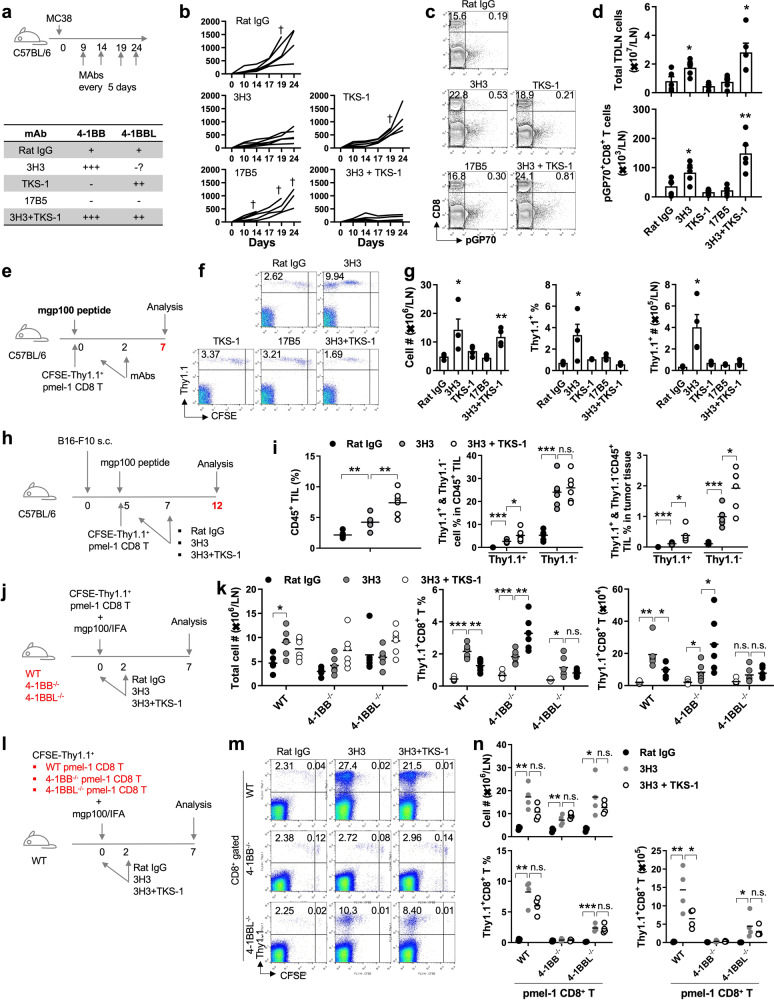
Anti-4-1BBL enhances 4-1BB-stimulated antitumor responses: (**a**) C57BL/6 mice were s.c. injected with MC38 tumor cells and i.p. administered 100 μg of mAbs on the indicated days. **b** Tumor growth rate. **c** iTDLN cells were counted and stained with pGP70-PE and anti-CD8β-PE-Cy5. **d** The absolute numbers of total and pGP70^+^CD8^+^ T cells in the inguinal TDLN were calculated. 4-1BBL promotes LN migration and tumor infiltration of activated pmel-1 CD8^+^ T cells: (**e**) Schematic diagram of the experiment. **f** Single-cell suspensions of inguinal LNs at Day 7 were counted and stained as described above. Gated CD8^+^ cells were plotted CFSE vs. Thy1.1. **g** Absolute numbers of inguinal LN cells and percentages and absolute numbers of pmel-1 Thy1.1^+^CD8^+^ T cells in inguinal LNs (*n* = 4 mice per experiment from three independent experiments). **h** Single-cell suspensions of tumor tissues at Day 12 were counted and stained with anti-CD8-PE, anti-Thy1.1-PE-Cy5, and anti-CD45-APC antibodies, and the percentages of CD45^+^ TILs and Thy1.1^+^ and Thy1.1^−^ cells in CD45^+^ TILs and tumor tissues were calculated (**i**). Peripheral 4-1BBL is required to induce migration of activated pmel-1 CD8^+^ T cells: (**j**) WT, 4-1BB^−/−^, and 4-1BBL^−/−^ C57BL/6 mice were i.v. injected with CFSE-labeled pme-1 Thy1.1^+^CD8^+^ T cells, s.c. immunized with 20 μg mgp100 peptide in IFA and i.p. administered 100 μg of rat IgG, 3H3, and 3H3 plus TKS-1 on Days 0 and 2. On Day 7, iTDLN cells were counted and stained with anti-CD8-PE and anti-Thy1.1-PE-Cy5 antibodies. Gated CD8^+^ cells were plotted CFSE vs. Thy1.1. **k** Absolute numbers of iTDLN cells. Percentages and absolute numbers of pmel-1 Thy1.1^+^CD8^+^ T cells in iTDLNs. (*n* = 6 mice per experiment from two independent experiments). Cellular division and migration of WT, 4-1BB^−/−^, and 4-1BBL^−/−^ pmel-1 CD8^+^ T cells: (**l**) C57BL/6 mice were i.v. injected with CFSE-labeled WT, 4-1BB^−/−^, and 4-1BBL^−/−^ pme-1 Thy1.1^+^CD8^+^ T cells and further immunized and administered with Abs as described. **m** Single-cell suspensions of iTDLNs at Day 7 were counted and stained with anti-CD8-PE and anti-Thy1.1-PE-Cy5 antibodies. Gated CD8^+^ cells were plotted CFSE vs. Thy1.1. **n** Absolute numbers of iTDLN cells and percentages and absolute numbers of pmel-1 Thy1.1^+^CD8^+^ T cells in the TDLN were calculated. (*n* = 4 mice per experiment from three independent experiments). Data are presented as the mean ± SD. *p* values were calculated using Student’s *t* test (*p < 0.05; **p < 0.01; ***p < 0.005)

To evaluate the effects of 3H3 and TKS-1 on self-tumor antigen-specific CD8^+^ T cells, pmel-1 Thy1.1^+^CD8^+^ T cells were activated with mouse gp100 (mgp100) peptide in vivo after they were transfected into C57BL/6 mice (Fig. 1e). At Day 7, pmel-1 CD8^+^ T cells in IgG-, TKS-1- or 17B5-treated mice showed comparable percentages and numbers, suggesting incomplete division in the draining lymph node (Fig. 1f, g). However, after >10 divisions, the percentages and absolute numbers of pmel-1 CD8^+^ T cells decreased in 3H3-treated mice and even further in 3H3 plus TSK-1-treated mice (Fig. 1f, g) compared with those on Day 5 (Supplementary Fig. 1). Notably, in a similar setting, the transcriptome analysis showed that TKS-1 treatment further upregulated the expression of genes involved in cell division, the type I IFN-related response, and T-cell activation, proliferation, and migration in 4-1BB-stimulated pmel-1 CD8^+^ T cells (Supplementary Fig. 2). After >10 divisions of CD8^+^ T cells, CD62L and CCR7 were downregulated, and sphingosine-1 phosphate receptor 1 was upregulated, enabling the migration of T cells from lymphoid organs [8–10]. Thus, we propose that 3H3 treatment enhances self-peptide-stimulated pmel-1 CD8^+^ T-cell division, possibly by amplifying TCR signaling [11, 12], and that TKS-1 treatment further accelerates the differentiation of 4-1BB-stimulated pmel-1 CD8^+^ T cells, leading to enhanced pmel-1 CD8^+^ T-cell migration from TDLNs.

In the B16-F10 melanoma mouse model (Fig. 1h), flow cytometry analysis 7 days after Ab treatment showed that 3H3 treatment increased, the proportion of CFSE-negative pmel-1 CD8^+^ T cells, whereas the addition of TKS-1 decreased the proportion of these cells in tumor-draining lymph nodes (Supplementary Fig. 3). Moreover, 3H3 treatment significantly increased the percentages of both endogenous Thy1.1^−^CD8^+^ tumor-infiltrating lymphocytes (TILs) and transferred pmel-1 Thy1.1^+^CD8^+^ TILs in the gated CD45^+^ cells, and coinjection of TKS-1 further significantly increased the percentage of pmel-1 Thy1.1^+^CD8^+^ TILs but not Thy1.1^−^CD8^+^ TILs (Fig. 1i). Moreover, due to the increase in the percentage of CD45^+^ TILs following 3H3 plus TKS-1 treatment, the percentages of pmel-1 Thy1.1^+^CD8^+^ TILs in tumor tissues were found to be significantly increased (Fig. 1i).

Reports suggest that 4-1BBL is expressed not only by APCs but also by activated T cells [3, 4]. However, it is not clear whether TKS-1 acts on APCs or T cells. To investigate, CFSE-labeled pmel-1 Thy1.1 + CD8 + T cells were transferred to WT, 4-1BB^−/−^, and 4-1BBL^−/−^ B6 mice, which were then immunized with mgp100 peptide and injected with mAbs (Fig. 1j). 3H3 treatment increased total TDLN cell numbers in WT but not 4-1BB^−/−^ and 4-1BBL^−/−^ B6 mice, while coadministration of TKS-1 with 3H3 decreased total TDLN cell numbers in WT mice and increased them in 4-1BB^−/−^ and 4-1BBL^−/−^ B6 mice (Fig. 1k). 3H3 treatment increased the pmel-1 Thy1.1^+^CD8^+^ T-cell percentage and number in all mouse types, while coinjection of TKS-1 decreased the percentage in WT mice, increased it in 4-1BB^−/−^ mice, and did not significantly change it in 4-1BBL^−/−^ mice (Fig. 1k, middle panel). 3H3 increased pmel-1 Thy1.1^+^CD8^+^ T cell numbers in WT and 4-1BB^−/−^ mice but not in 4-1BBL^−/−^ mice, while coadministration of TKS-1 reduced pmel-1 Thy1.1^+^CD8^+^ T cell numbers in WT mice, increased them in 4-1BB^−/−^ mice, and did not significantly affect them in 4-1BBL^−/−^ mice (Fig. 1k; right panel). Peripheral 4-1BB deficiency unexpectedly increased pmel-1 CD8^+^ T cell numbers following coinjection of 3H3 and TKS-1, but the cause of this T-cell hyperresponsiveness was not clear. Nonetheless, peripheral 4-1BBL deficiency reduced the number of 4-1BB-stimulated pmel-1 Thy1.1^+^CD8^+^ T cells and impaired the effects of TKS-1, indicating that peripheral 4-1BBL enhances the migration of 4-1BB-stimulated pmel-1 CD8^+^ T cells following TKS-1 treatment.

Next, we investigated whether 4-1BB or 4-1BBL deficiency in pmel-1 CD8^+^ T cells affects the migration of 4-1BB-stimulated pmel-1 CD8^+^ T cells from the lymph nodes after TKS-1 treatment. CFSE-labeled pmel-1 Thy1.1^+^CD8^+^ T cells from WT, 4-1BB^−/−^, and 4-1BBL^−/−^ mice were transferred to WT B6 mice (Fig. 1l). The recipient mice were immunized with mgp100 peptide and injected with mAbs as previously described. The analysis confirmed the previous findings on total cell numbers, percentages, and pmel-1 CD8 + T-cell counts in B6 recipient mice treated with 3H3 or 3H3 plus TKS-1 after transferring WT pmel-1 CD8^+^ T cells (Fig. 1j, k). However, 4-1BB^−/−^ pmel-1 CD8^+^ T cells were hardly detected in TDLNs due to their low survival rates, resulting in the loss of the effects of 3H3 or 3H3 plus TKS-1 (Fig. 1m, n; [13, 14]). After transferring 4-1BBL^−/−^ pmel-1 CD8^+^ T cells to WT mice, 3H3 alone significantly increased TLDN cell numbers and the percentages and absolute counts of pmel-1 CD8^+^ T cells, while coinjection of TKS-1 completely abrogated its effects (Fig. 1n). Furthermore, the absolute count of 4-1BBL^−/−^ pmel-1 CD8^+^ T cells was one-third of that of WT pmel-1 CD8^+^ T cells in 3H3-treated mice (Fig. 1n; last panel).

Coculturing increasing ratios of WT CD8^+^ T cells with WT and 4-1BBL^−/−^ Thy1.1^+^CD8^+^ T cells resulted in a minimal effect on the survival and proliferation of WT Thy1.1^+^CD8^+^ T cells but a lower overall survival of Thy1.1^+^CD8^+^ T cells when T cells lacked 4-1BBL (Supplementary Fig. 4), suggesting that the trans interaction of 4-1BB and 4-1BBL between CD8^+^ T cells has a negligible effect on their survival and that the *cis*-interaction of 4-1BB and 4-1BBL in CD8^+^ T cells is necessary to support activated CD8^+^ T-cell survival.

Overall, our results suggest that the expression of endogenous 4-1BB in CD8^+^ T cells is indispensable for their survival during cellular division and that the expression of endogenous 4-1BBL is also necessary to maintain the survival of dividing CD8^+^ T cells and to enhance the migration of 4-1BB-stimulated CD8^+^ T cells after TKS-1 treatment.

## Supplementary information

Supplementary Fig 1

Supplementary Fig 2

Supplementary Fig 3

Supplementary Fig 4

Supplementary figure legend

## Data Availability

The RNA-seq data have been deposited in the ArrayExpress database at EMBL-EBI under the accession number E-MTAB-13053.
