# Supplementary figures and images for: 4-1BB-4-1BBL cis-interaction contributes to the survival of self-reactive CD8+ T cell
Source: Cell Mol Immunol. 2023 Jun 26;20(9):1077–80. doi: 10.1038/s41423-023-01056-3 (PMC10468488; doi:10.1038/s41423-023-01056-3)

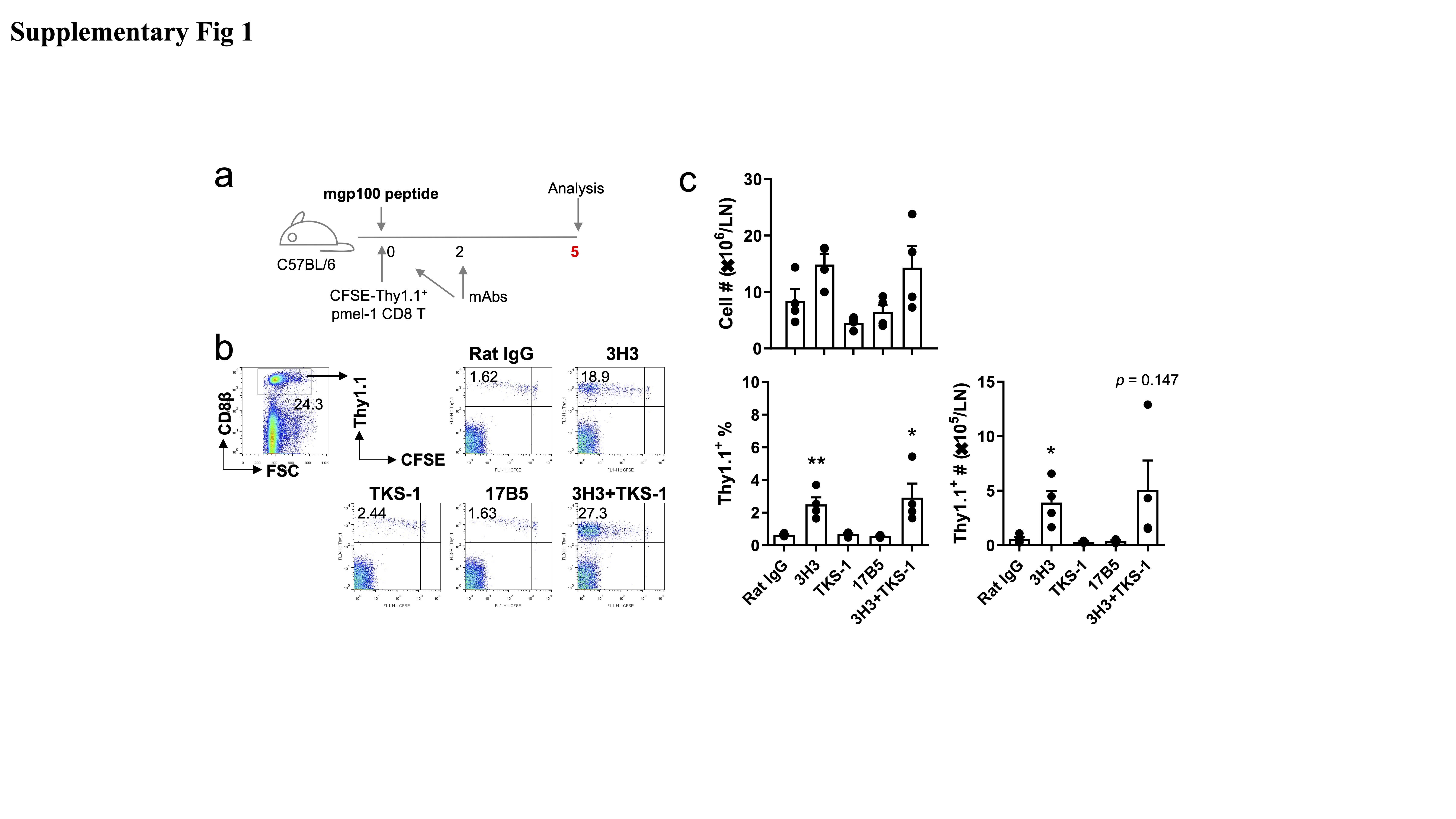

Supplement: Supplementary file 1 — Supplementary Fig 1 [file 41423_2023_1056_MOESM1_ESM.jpg]

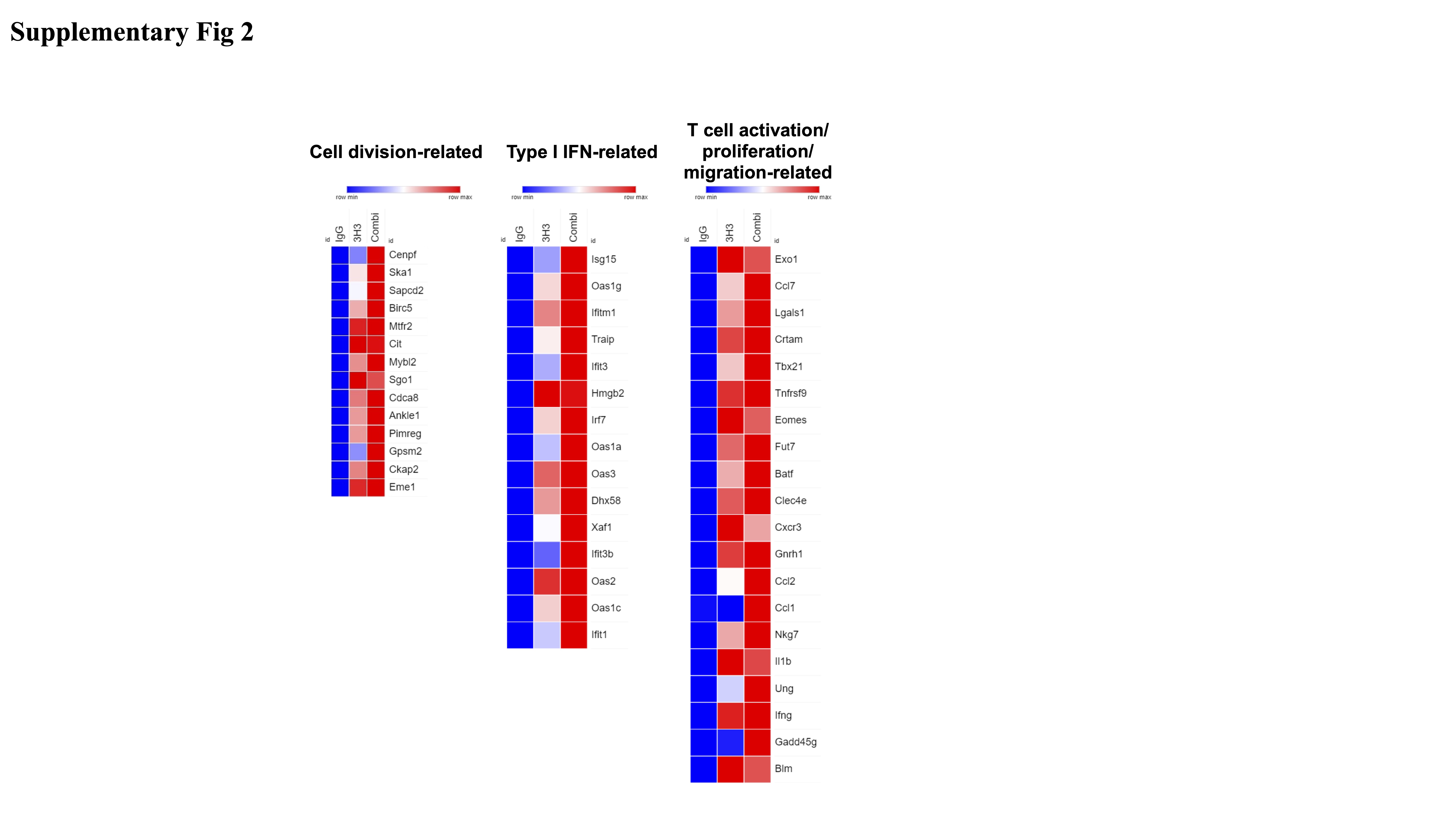

Supplement: Supplementary file 2 — Supplementary Fig 2 [file 41423_2023_1056_MOESM2_ESM.jpg]

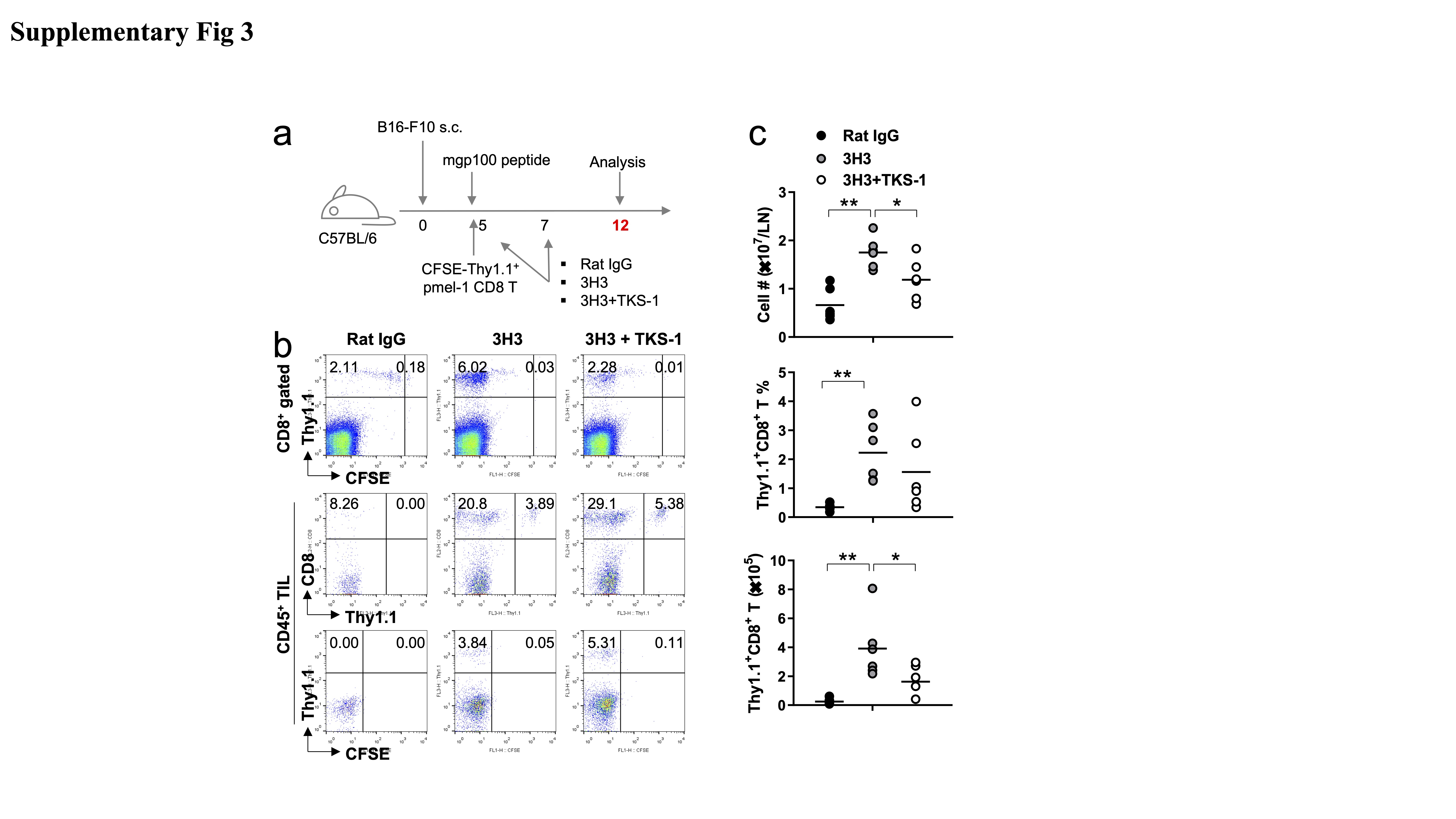

Supplement: Supplementary file 3 — Supplementary Fig 3 [file 41423_2023_1056_MOESM3_ESM.jpg]

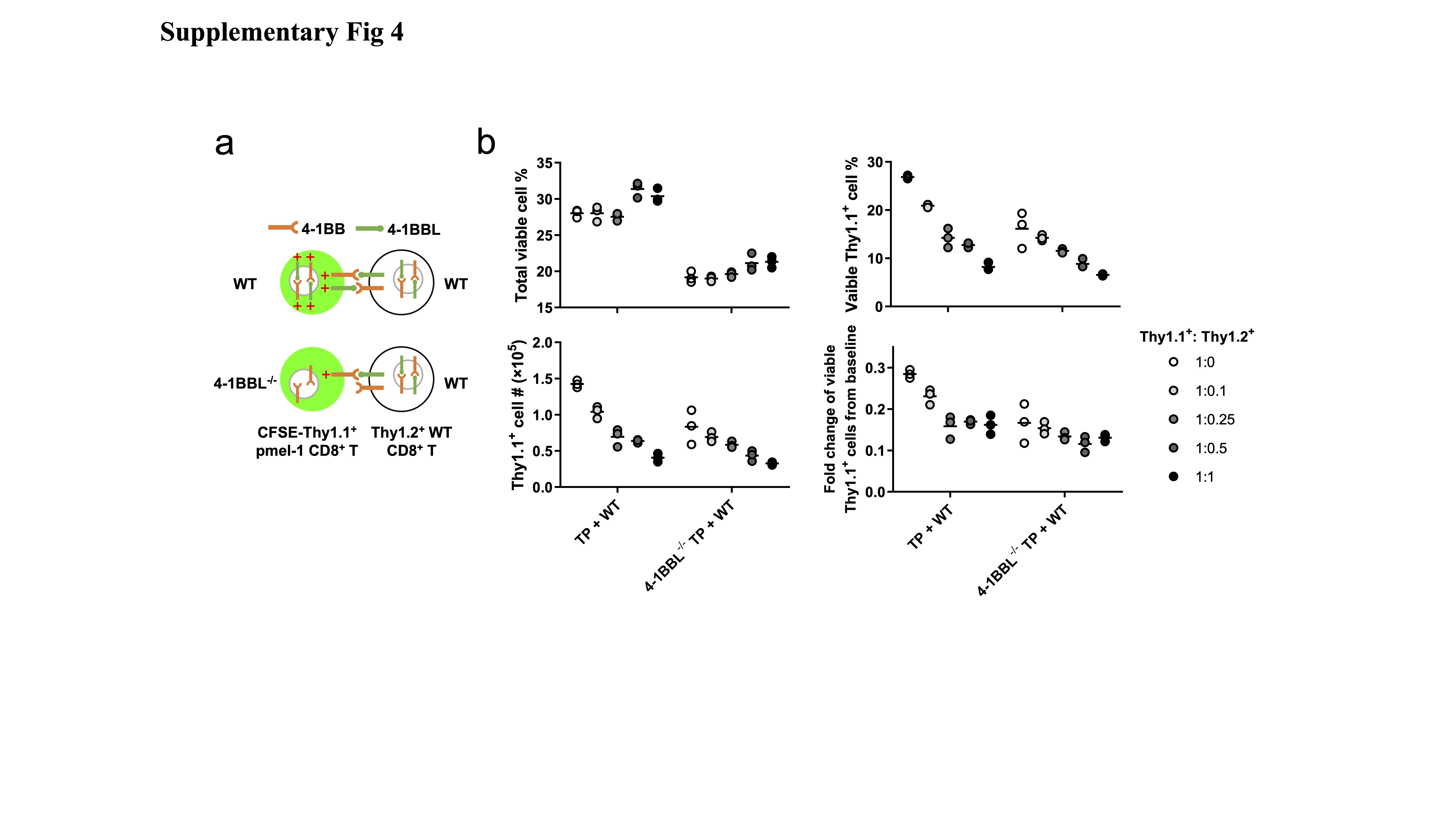

Supplement: Supplementary file 4 — Supplementary Fig 4 [file 41423_2023_1056_MOESM4_ESM.jpg]
