# Supplementary material for: 4-1BB-4-1BBL cis-interaction contributes to the survival of self-reactive CD8+ T cell
Source: Cell Mol Immunol. 2023 Jun 26;20(9):1077–80. doi: 10.1038/s41423-023-01056-3 (PMC10468488; doi:10.1038/s41423-023-01056-3)
Supplement: Supplementary file 5 — Supplementary figure legend [file 41423_2023_1056_MOESM5_ESM.docx]

**Supplementary Fig. 1. Pmel-1 CD8^+^ T cells stimulated with mgp100 peptide *in vivo* are maximally divided by 3H3 alone and 3H3 + TKS treatment.** **(a)** C57BL/6 mice were i.v. injected with CFSE-labeled pme-1 Thy1.1^+^CD8^+^ T cells, s.c. immunized with 20 μg mgp100 peptide in IFA and i.p. administered 100 μg of rat IgG, 3H3, 17B5, TKS-1, and 3H3 plus TKS-1 at Days 0 and 2. **(b)** Single-cell suspensions of inguinal LNs at Day 7 were counted and stained with anti-CD8-PE and anti-Thy1.1-PE-Cy5 antibodies. Gated CD8^+^ cells were plotted CFSE vs. Thy1.1. **(c)** Absolute numbers of inguinal LN cells and percentages and absolute numbers of pmel-1 Thy1.1^+^CD8^+^ T cells in inguinal LNs. Data are from three independent experiments with 4 mice per experiment. Student’s *t* test was performed in c, and data are presented as the mean ± SD (**p* < 0.05; ***p* < 0.01).

**Supplementary Fig. 2. Transcriptome analysis of pmel-1 CD8^+^ T cells treated with rat IgG, 3H3, and 3H3 plus TKS-1.** B6 mice were i.v. injected with 5 × 10^5^ pme-1 Thy1.1^+^CD8^+^ T cells, immunized with mgp100 peptide emulsified in IFA, and i.p. administered 100 μg of rat IgG, 3H3 or 3H3 plus TKS-1 on Days 0 and 2. Five days after cell transfer, inguinal LNs were collected from each group of mice, and Thy1.1^+^ cells were isolated using Thy1.1 microbeads. Total RNA was isolated from pmel-1 Thy1.1^+^CD8^+^ T cells using TRIzol reagent (Invitrogen). RNA purity and integrity were evaluated using an ND-1000 spectrophotometer (NanoDrop, Wilmington, USA) and an Agilent 2100 Bioanalyzer (Agilent Technologies, CA, USA), respectively. QC-passed RNA samples were used to perform RNA sequencing (Theragen Bio, Seoul, Korea).

**Supplementary Fig. 3. The engagement of CD8^+^ T cells with 4-1BBL increases the tumor infiltration of pmel-1 CD8^+^ T cells.** **(a)** C57BL/6 mice were s.c. injected with B16-F10 melanoma cells. Five days after the tumor injection, the mice were i.v. injected with CFSE-labeled pme-1 Thy1.1^+^CD8^+^ T cells and were further immunized and administered Abs as indicated. **(b)** Single-cell suspensions of inguinal TDLNs and tumor tissues at Day 12 were counted and stained with anti-CD8-PE, anti-Thy1.1-PE-Cy5, and anti-CD45-APC antibodies. CD45^+^CD8^+^ cells were gated for TDLN and plotted CFSE vs. Thy1.1. CD45^+^ cells were gated for tumor tissues, and CD8 vs. Thy1.1 or CFSE vs. Thy1.1 was plotted. **(c)** Absolute numbers of inguinal TDLN cells and percentages and absolute numbers of pmel-1 Thy1.1^+^CD8^+^ T cells in an inguinal LN were calculated. Percentages of CD45^+^ TILs and percentages of Thy1.1^+^ and Thy1.1^-^ cells in CD45^+^ TILs and tumor tissues were calculated from **(b)**. Data are from two independent experiments with 6 mice per experiment. Student’s *t* test was performed in c, and data are presented as the mean ± SD (**p* < 0.05; ***p* < 0.01; ****p* < 0.005).

**Supplementary Fig. 4. Trans interaction of 4-1BB and 4-1BBL between CD8^+^ T cells has a minimal effect on enhancing the survival of CD8^+^ T cells.** (**a**) CFSE-labeled CD8^+^ T cells from WT and 4-1BBL^-/-^ pmel-1 Thy1.1^+^ Tg mice were mixed with unlabeled CD8^+^ T cells from Thy1.2^+^ WT B6 mice. Consequently, a cis interaction between 4-1BB and 4-1BBL did not occur in 4-1BBL^-/-^ pmel-1 CD8^+^ T cells. To avoid any confounding effects of increased cell density on T-cell proliferation, we adjusted the total cell number to a fixed value of 5 × 10^5^ cells per well while also increasing the ratio of unlabeled CD8^+^ T cells. After coculturing the cells in the presence of anti-CD3 mAb for three days, we analyzed the level of proliferation and survival among the T-cell populations using flow cytometry. The cultured cells were counted and stained with anti-CD8-PE and anti-Thy1.1-PE-Cy5 antibodies. Gated CD8^+^ cells were plotted CFSE vs. Thy1.1. (**b)** Total viable cells were determined with an automated cell counter. The percentages and absolute numbers of viable Thy1.1^+^ cells were calculated. Fold changes in viable Thy1.1^+^CD8^+^ T cells from baseline were calculated. Data are from two independent experiments (n=3).
